# CdS quantum dots-based immunoassay combined with particle imprinted polymer technology and laser ablation ICP-MS as a versatile tool for protein detection

**DOI:** 10.1038/s41598-019-48290-2

**Published:** 2019-08-14

**Authors:** Tereza Vaneckova, Jaroslava Bezdekova, Michaela Tvrdonova, Marcela Vlcnovska, Veronika Novotna, Jan Neuman, Aneta Stossova, Viktor Kanicky, Vojtech Adam, Marketa Vaculovicova, Tomas Vaculovic

**Affiliations:** 1 Department of Chemistry and Biochemistry, Mendel University in Brno, Zemedelska 1, CZ-613 00 Brno, Czech Republic; 2 Central European Institute of Technology, Brno University of Technology, Purkynova 123, CZ-612 00 Brno, Czech Republic; 3 NenoVision s.r.o., Purkynova 649/127, CZ-612 00 Brno, Czech Republic; 4 Department of Chemistry, Masaryk University, Kamenice 753/5, CZ-625 00 Brno, Czech Republic

**Keywords:** Biochemistry, Immunochemistry

## Abstract

For the first time, the combination of molecularly imprinted polymer (MIP) technology with laser ablation inductively coupled plasma mass spectrometry (LA-ICP-MS) is presented with focus on an optimization of the LA-ICP-MS parameters such as laser beam diameter, laser beam fluence, and scan speed using CdS quantum dots (QDs) as a template and dopamine as a functional monomer. A non-covalent imprinting approach was employed in this study due to the simplicity of preparation. Simple oxidative polymerization of the dopamine that creates the self-assembly monolayer seems to be an ideal choice. The QDs prepared by UV light irradiation synthesis were stabilized by using mercaptosuccinic acid. Formation of a complex of QD-antibody and QD-antibody-antigen was verified by using capillary electrophoresis with laser-induced fluorescence detection. QDs and antibody were connected together *via* an affinity peptide linker. LA-ICP-MS was employed as a proof-of-concept for detection method of two types of immunoassay: 1) antigen extracted from the sample by MIP and subsequently overlaid/immunoreacted by QD-labelled antibodies, 2) complex of antigen, antibody, and QD formed in the sample and subsequently extracted by MIP. The first approach provided higher sensitivity (MIP/NIP), however, the second demonstrated higher selectivity. A mixture of proteins with size in range 10–250 kDa was used as a model sample to demonstrate the capability of both approaches for detection of IgG in a complex sample.

## Introduction

The technology of imprinted polymers allows for a selective recognition of targeted analyte. It is based on a formation of an analyte recognition surface that is created after polymerization of a mixture of analyte and suitable monomer^1,2^. Molecules or ions can be imprinted into polymers then molecularly or ion imprinted polymers (MIP or IIP, respectively) are formed. Nowadays, MIPs are used as specific selectors for pre-concentration of broad groups of analytes in food^3,4^, beverages^5,6^, water^7^, soil^8^, and biological samples (e.g. blood plasma^9^). The main benefit of this technology is in its variability. Compared to conventional immunoassay, the use of MIP technology is applicable for analytes for which the antibodies are unavailable. Moreover, MIPs can be employed for a broad range of analytes (from ions to microorganisms) and take advantage of various surface arrangements (well-plates, microscopic slides, mass spectrometric targets, electrode and/or even nanoparticle surfaces). The monomer/polymer system can be also varied to provide not only suitable functional groups increasing the specificity of the interaction with the target analyte, but also suitable polymerization conditions^10^. Moreover, a number of detection techniques (e.g., fluorescence spectrometry or microscopy, mass spectrometry, and/or quartz crystal microbalance) can be selected. The most commonly employed type of detection method is probably the quartz crystal microbalance^11^, however extremely sensitive techniques such as inductively coupled plasma mass spectrometry (ICP-MS) may provide benefits for certain applications^12^.

One of the polymers used for MIPs is polydopamine offering numerous benefits compared methacrylate and its derivatives^13^. Polydopamine was used for the first time as a surface coating in 2007 by Lee *et al*.^14^ and since then, the benefits of this material have been exploited in various fields including molecular imprinting^15^. Simple oxidative polymerization of dopamine is involved during the formation of the self-assembly layer^16^. Even though the detail mechanism of the polymerization is still not completely clear, it has been widely to create MIP on surfaces such as particles^17–19^, monolithic columns^20^ and/or carbon nanomaterials^15,21^.

MIPs have found its application in biochemistry and bioanalysis providing an alternative to antibodies used in immunoassays. Standard immunoassays are commonly visualized by optical techniques, such as fluorescence^22,23^, chemi(bio)luminescence^24^, colorimetric detection^25–28^ or more recently introduced photothermal method^29^, however in some cases, a more sensitive detection is required. For such applications, metal-labelled antibodies in combination with laser ablation inductively coupled plasma mass spectrometry (LA-ICP-MS) is the method of choice.

Inductively coupled plasma mass spectrometry (ICP-MS) is a well-established analytical method for multi-elemental analysis for elements at trace- and ultra-trace levels. It is outstanding mainly due to the high sensitivity independent of the molecular structure of the analyte, wide linear dynamic range and due to excellent multi-element capabilities. In combination with laser ablation sample introduction, ICP-MS has also been used to image the distribution of metallic nanoparticles (Au, Ag) in single biological cells^30^.

As it was shown, NP-labelled antibodies in combination with ICP-MS used in dot-blot analysis are providing 4 orders of magnitude improved detection limits compared to standard metal-based labelling approach^31^. The idea of MIP-based technology has been employed in combination with nanoparticles (NPs) made of Fe_3_O_4_^32,33^, gold NPs^34^, CuO NPs^35^, EuS NPs^36^, quantum dots^37–44^ and upconversion NPs^45,46^.

For the first time, the combination of LA-ICP-MS with the technology of imprinted polymers is presented by application on the immunoassay using CdS quantum dots-labelled antibodies. By the technology of imprinted polymers (polydopamine), the analyte is selectively captured, and antibodies tagged by semiconductor NPs provided a high sensitivity signal. This proof-of-concept study combining for the first time the advantages of MIP and LA-ICP-MS is presented with special attention paid to optimization of the LA-ICP-MS method in terms of laser ablation parameters.

## Experimental

### Materials

Natural Mouse IgG protein (ab198772) and Goat Anti-Mouse IgG H&L (ab6708) were purchased from AbCam (Cambridge, UK). HWRGWVC peptide was obtained from Clonestar Peptide Services, s.r.o., (Brno, Czech Republic). Dopamine hydrochloride, Mercaptosuccinic acid (MSA), Cadmium (Cd), Trizma base, Na_2_HPO_4_ and NaH_2_PO_4_ were purchased from Sigma-Aldrich (St. Louis, MO, USA) in ACS purity. 2-propanol and sodium tetraborate decahydrate were obtained from Thermo Fisher Scientific (Waltham, MA, USA).

### Preparation of quantum dots

CdS QDs were prepared by mixing 50 μl Cd solution (3 mM) with 25 μl 0.1 M sodium phosphate buffer (Na_2_HPO_4_/NaH_2_PO_4_) pH 7 and 25 μl MSA (2.4 mg·ml^−1^) all 96 wells of UV-transparent well-plate. Then the plate was placed in UV trans-illuminator and was irradiated (λ = 254 nm) for 10 minutes. Resulted QDs were precipitated by 2-propanol (mixed 1:2 QDs:2-propanol, shaking for 10 minutes, and centrifugation for 10 min at 9000 rpm). Supernatant was removed and pellet was dried by solvent evaporation at 50 °C^47^.

### Characterization of prepared QDs

The prepared QDs were characterized by dynamic light scattering measuring the size, polydispersity, and zeta-potential using Zetasizer Nano ZS (Malvern, UK) according to the manufacturer’s instruction. Moreover, fluorescence and absorbance spectroscopic characterization was performed using Plate reader Infinite 200 PRO (Tecan, Switzerland). Wavelength 350 nm was used as an excitation radiation and the fluorescence scan was measured within the range from 400 to 700 nm per 2-nm steps. The detector gain was set to 100. The samples were placed in UV-transparent 96 well microplate with flat bottom by CoStar (Corning, USA). To each well 50 μL of the sample was placed. All measurements were performed at 25 °C.

### Preparation of QD-antibody conjugates

1.7 µl of solution of conjugation peptide (HWRGWVC) abbreviated as HWR peptide (1.25 mg ml^−1^) was added to 25 µl of CdS QDs (0.125 mg ml^−1^) capped with MSA and the mixture was incubated using Thermomixer 5355 (Eppendorf, Germany) at 45 °C, 600 rpm for 1 hour. Subsequently, 1.6 µl of antibody (AB, 1 mg ml^−1^) was added to the QDs-HWR peptide conjugate. The mixture was again reacted using the Thermomixer at 21 °C, 600 rpm for 1 hour. Then the conjugate QD-AB linked *via* HWR peptide was ready to use^31^.

### Characterization of QD-AB conjugates by capillary electrophoresis with laser-induced fluorescence detection (CE-LIF)

Prepared conjugates were characterized by CE-LIF using Beckman PACE/MDQ with excitation using light emitting diode with emission wavelength of 395 nm. An uncoated fused silica capillary with total length of 47 cm and effective length of 40 cm was used. The internal diameter of the capillary was 75 μm. 20 mM sodium borate buffer (pH = 9) was used as a background electrolyte and the separation was carried out using 20 kV with hydrodynamic injection by 5 psi for 5 s^31^.

### Preparation of imprinted surface

The mixture (1 μl) of dopamine (5 mg ml^−1^ in tris buffer pH 8.5) and the template (quantum dot-antibody = QD-AB, or quantum dot-antibody-antigen = QD-AB-AG complex) in a ratio of 1:1 was polymerized (overnight at room temperature) on the surface of the glass microscopic slide to form a thin film of polydopamine with specific cavities selective to analyte (QD-AB or QD-AB-AG complex). Non-imprinted layer (NIP) was used as a control. The MIP/NIP spot was in average 2 mm in diameter and all experiments (MIP as well as NIP) were performed in triplicates. The final concentration of the QD and AB in the template was therefore 0.055 mg ml^−1^ and 0.028 mg ml^−1^, respectively. Then the template was removed by washing for five-times with 10 μl of acetic acid (10%) and twice with 10 μl of MilliQ water. The surface of polymer was blocked by using 5 μl mixture of milk (10%) and 0.1 M phosphate buffer, pH 7 (90%) for 10 minutes. Subsequently, the surface was washed three-times with 10 μl of MilliQ water. Next, the sample (1 μl) containing the analyte was dosed on the imprinted surface and after 1 hour of interaction, the surface was rinsed three-times by MilliQ water (10 μl). It was necessary to prepare NIP, formed from polydopamine without the presence of the template. It served as a blank.

### Imprinted surface characterization

The sample surface was analyzed using SEM LYRA3 (TESCAN, Czech Republic) with integrated AFM LiteScope (NenoVision, Czech Republic). Correlative Probe and Electron Microscopy (CPEM)^48^ was used for the surface analysis allowing simultaneous acquisition of SEM and AFM images at the same place in the same coordinate system. The SEM contrast is sensitive to the sample composition, while the AFM provides real surface topography. The accelerating voltage of 5 kV, beam current of 13 pA and SE detector was used for SEM imaging. The self-sensing Akiyama probe in tapping mode was used for the AFM measurement.

### LA-ICP-MS

The analysis of MIP was performed by LA-ICP-MS setup that consists of LA system UP213 (NewWave Research, USA) emitting laser radiation with a wavelength of 213 nm with a pulse width of 4.2 ns. The ablated material was carried out from an ablation cell by a flow of a He (1.0 l/min) into ICP-MS Agilent 7500CE (Agilent Technologies, Japan) with quadrupole analyzer. The optimized laser ablation parameters used for MIP and NIP analysis were following: laser beam diameter of 250 μm, the repetition rate of 10 Hz, laser beam fluence of 2 J/cm^2^, the scan speed of 2000 μm/s and distance between individual lines of 210 μm. The signal arising from CdS QD was monitored via isotope ^111^Cd. The whole spot of the sample was ablated by line patterns and signals of monitored isotopes were observed. The limit of quantification for signal intensity is calculated according to 10- fold of standard deviation of the gas level (without ablation). All intensities below LOQ were replaced by zero. Then sum intensity across the whole spot was calculated.

Limits of detection was calculated according to 3σ: LOD = (3 × Standard deviation of blank)/slope.

As a blank value, three spots of NIPs were measured and the standard deviation was then calculated from the sum of intensities of each one. The slope value was obtained from the sum of intensities of MIP (2.5 μg of IgG).

### Data processing

First, the threshold value for ^111^Cd signal was calculated. It was the lowest measured intensity taken into count for data evaluation. It was calculated as a sum of the average blank level of ^111^Cd signal and 10 fold standard deviation of a blank level. Blank level corresponding to intensities measured in carrier gas before ablation start. Time resolved signal of ^111^Cd was obtained for each spot and intensities, lower than the threshold value, were removed. Then total sum of intensities of ^111^Cd was calculated for each spot. The threshold value caused that the sum of intensities did not grow due to background level intensities.

## Results

### Characterization of prepared QDs

For the characterization of CdS QDs their size, polydispersity, ζ-potential, and absorption and emission spectra were measured. From the DLS analysis follows that the average particle size is 18 nm and the negative value of ζ-potential (−41 mV) indicated that the prepared QDs were stable and did not aggregate. This measurement is supported by their emission spectrum showing the emission maximum to be 548 nm. Size distribution, DLS data as well as TEM micrographs are shown in S1–S3.

### CE-LIF of QD-conjugates

**Figure 1:**
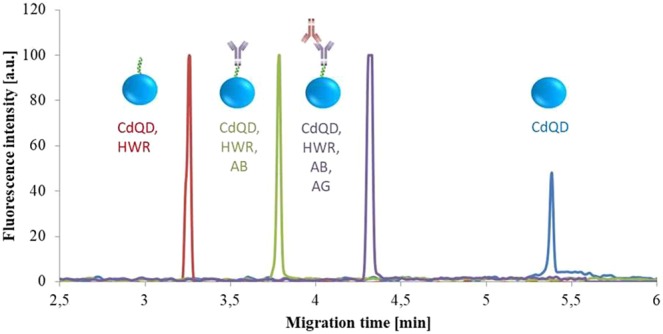
Electropherogram recording consequent complex formation by CE-LIF. Fluorescence excitation at 395 nm, total length 47 cm, effective length 40 cm, internal diameter 75 μm, electrolyte - 20 mM sodium borate buffer (pH = 9), separation voltage - 20 kV, injection - 5 psi for 5 s.

To verify the formation of the conjugates, CE-LIF was used. QDs, QDs-HWR, QDs-HWR-AB, and QDs-HWR-AB-AG were measured to confirm that each step of conjugation was successful. As can be seen from the Fig. 1, formation of various species was confirmed. The CdS QDs have negative charge as found by measurement of ζ-potential. Hence they have the highest migration time (5.38 minutes). HWR peptide sequence contains several positively charged amino acids and therefore, the conjugate of QD with HWR exhibited positive charge and its migration time decreased (3.26 minutes). The migration time of QD-HWR-AB complex was 3.78 minutes due to the increase in the size as well as increase of the negative charge. Similarly, when the complex QD-HWR-AB-AG was created (after addition of AG in total concentration 0.026 mg ml^−1^), further increase in migration time was observed (4.32 minutes). Moreover, significant increase in the fluorescence intensity is observed when the HWR peptide is connected to CdS QDs. It is supposed that the increase relates to the fluorescence resonance energy transfer occurring within the formed complex. HWR peptide contains 1 molecule of histidine with an aromatic ring responsible for absorption maximum at 390 nm^49^. Hence the light supplied by the excitation LED was absorbed by the histidine and transferred to the connected QD. This is believed to lead to the fluorescence signal enhancement. However this was not the aim of this study and therefore, it was not further investigated in detail.

### Optimization of MIP

A non-covalent imprinting approach was employed in this study due to the simplicity of preparation. The polymeric layer was created on the surface of a glass slide according to the method published previously by our group^9^. The concentration of 16 mM (2.5 mg ml^−1^) dopamine hydrochloride was determined as the most effective considering the imprinting ratio (data not shown).

### Optimization of ablation parameters

**Figure 2:**
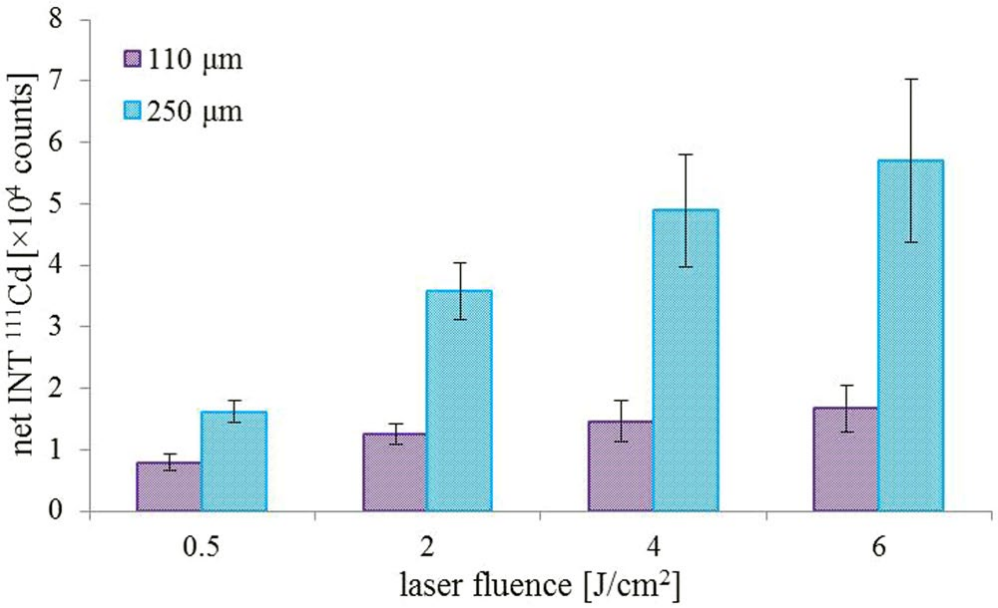
Influence of laser beam diameters (110 and 250 μm) and fluence (0.5, 2, 4, and 6 J/cm^2^) on intensity of ^111^Cd signal.

Laser ablation parameters as the laser beam fluence, laser spot size and scan speed were optimized to get a combination of both parameters with high sensitivity and high stability (low relative standard deviation - RSD) of the measured signal of ^111^Cd. For all optimization, the slide was covered by a layer of polydopamine surface imprinted with CdS QDs with a diameter of 18 nm. First, optimization of laser beam fluence and laser spot size was carried out. Laser ablation system operates in 2 modes of laser beam focusing: I) Imaging mode – the laser spot size selected by using suitable aperture and II) Focused mode – the laser spot size selected by moving the lens. In the case of the imaging mode, the laser beam energy was more homogeneous across the laser beam cross section than in case of the Focused mode. To get the highest amount of the ablated material the largest available laser beam diameters for each focusing mode were tested – 110 (imaging) and 250 μm (focused). The laser beam fluence was changed in a range from 0.5 to 6.0 J/cm^2^. Under optimal laser beam fluence and laser spot size, the optimization of scan speed was done. Each combination of laser spot size and laser beam fluence was done as a line scan in triplicates on different places on the slide. The arithmetic average and relative standard deviations were calculated. The results of the optimization of laser beam spot size and fluence are summarized in Fig. 2 The pink and blue columns represent the laser beam diameters of 110 and 250 μm, respectively. As expected, the 250 μm laser beam spot provided significantly higher intensities compared to the 110 μm laser beam. In the case of the signal stability, the results obtained by 250 μm laser beam spot exhibited lower RSD compared to 110 μm laser beam spot. Hence, the laser beam spot of 250 μm was selected as optimal. Once the laser beam fluence was optimized, the largest intensities were achieved for 4 and 6 J/cm^2^. However, the lower stability of the signal was obtained. Therefore, the laser beam fluence of 2 J/cm^2^ was used as a compromise between the signal sensitivity and stability.

Subsequently, the optimal scan speed was searched. It was found that scan speed affected neither the sensitivity nor the stability of the ^111^Cd signal. The RSD of the signal intensity varied from 10 to 15% for all 4 tested scan speeds (300, 800, 1200 and 2000 μm/s). Hence, the criterion for selection of scan speed was the duration of the analysis. When the scan speed of 2000 μm/s was applied, the overall duration of the analysis was about 23 s.

### MIP-immunoassay design

**Figure 3:**
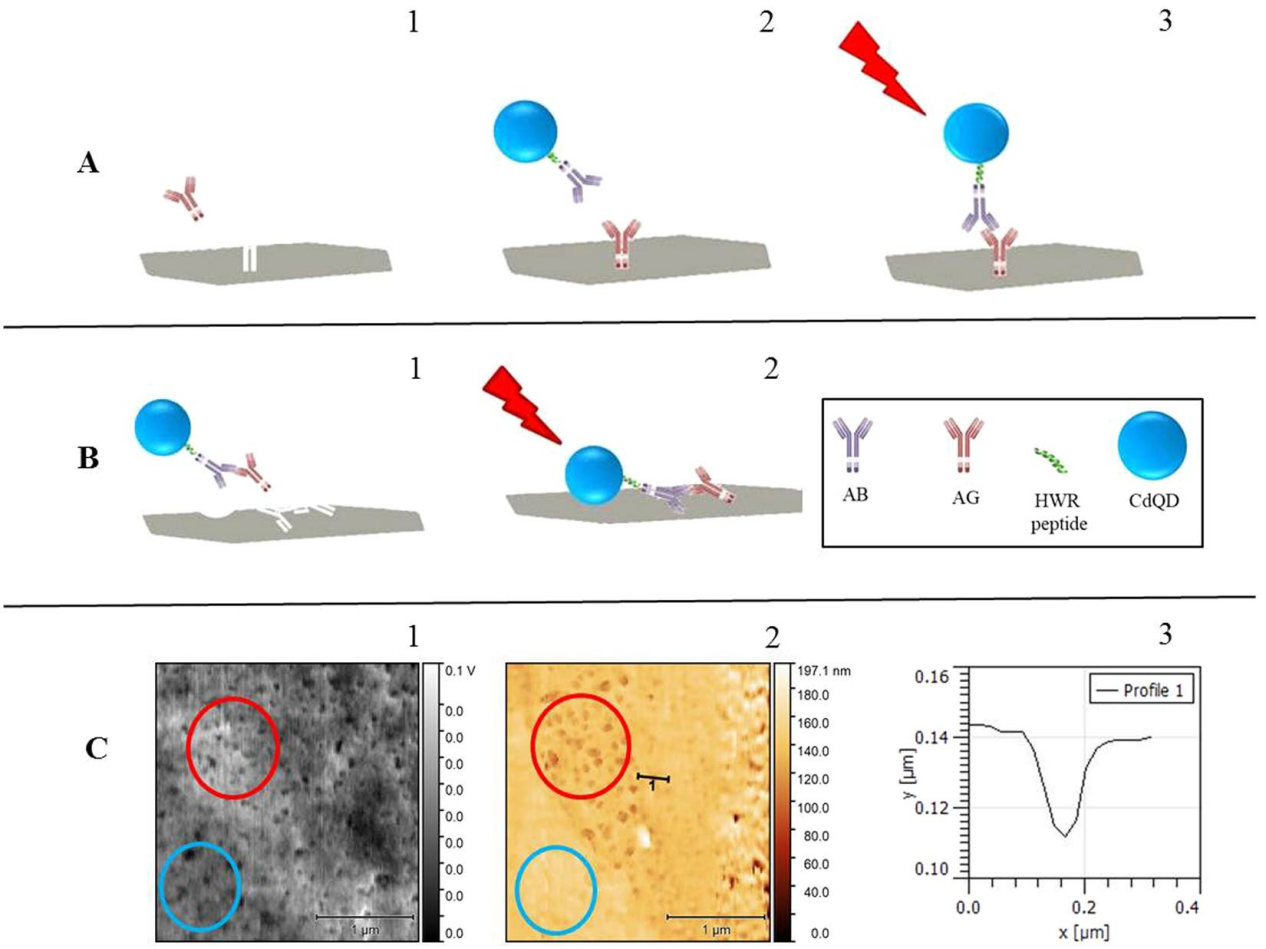
Schematic representation of two concepts of immunoassay combining QD-labelled antibodies and MIP technology. (**A**) 1-imprinting of the AG, 2-extraction of the AG from the sample by MIP, overlay with QD-AB conjugate, 3-interaction of the conjugate with the extracted AG from the sample, removal of the unreacted conjugate. (**B**) 1 - imprinting of the complex QD-AB-AG, 2 - QD-AB-AG formed in the sample after addition of QD-AB conjugate into the sample solution and extraction of QD-AB-AG complex from the sample by MIP for removal of interferents from the sample. (**C**) Correlative Probe and Electron Microscopy (CPEM) imaging of MIP layer with imprinted QD-AB conjugate. 1 – SEM image, 2 - AFM image of the same area and sample in the same coordinate system. 3 – profile of a well formed due to the imprinting process.

Two approaches towards the MIP-immunoassay design (schematically shown in Fig. 3A,B) were tested. In the first concept (concept labelled A), only the AG was used as a template and therefore, the MIP selective only for AG was prepared. After that, AG was selectively isolated from the sample and subsequently, the MIP surface (with AG extracted from the sample) was overlaid by the QD-AB conjugate.

In the second approach (concept labelled B), the whole complex QD-AB-AG was imprinted (used as template) and during the analysis, this complex was formed directly within the sample and subsequently extracted by MIP.

In Fig. 3C, the surface visualization obtained by Correlative Probe and Electron Microscopy (CPEM) is shown. SEM image (1) and AFM image (2) were obtained simultaneously from the same region of the sample. Mainly two regions of the surface are of our interest (red and blue circle). Even though SEM image shows similar contrast of both regions, AFM imaging clearly confirms differences between the flat surface (blue circle) and wells formed due to the imprinting process (red circle). Profile of one of the wells is shown in (3).

**Figure 4:**
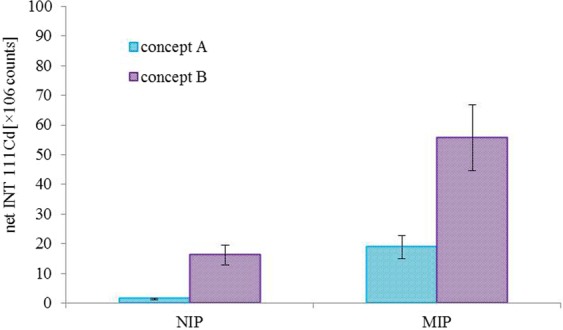
ICP-MS signal intensities (MIPs and NIPs) obtained by analysis of IgG standard sample by assay concepts A and B described in Fig. 3.

As shown in Fig. 4, significant differences between MIP and NIP were detected in both concepts. The MIP/NIP signal ratio was 13.5 and 3.4 for concept A and concept B, respectively. This result, however demonstrates the concept A to be less influenced by the non-specific adsorption of the sample components on the polymeric layer (NIP signal).

Lower intensities (both MIP and NIP) are observed in concept A compared to concept B. This is probably due to the fact that when the concept A is applied, the antigen only is imprinted, but its orientation in the polydopamine layer may not always be correct; that means, when the antigen is oriented so that the interaction site is not accessible for the QD-labelled antibody, the antigen-antibody interaction is prevented.

On the other hand, the concept B is providing 2.9-fold higher MIP signal compared to concept A suggesting better sensitivity of this approach. The significantly higher NIP signal (11.6-fold) of B concept compared to approach A is probably given by the fact that some mole-cules of AA-QD complex are more prone to non-specific adsorption.

The LODs of both concepts were calculated according to 3 σ: concept A – 4.2 μg, concept B – 1.6 μg.

NIP is prepared under identical conditions and also with identical construction as MIP. The difference is that NIP is prepared without the presence of the template. It is taken as an indicator of non-specific binding. The NIP has functional groups randomly arranged on its surface that interact with analyte and cause to some extent bindings. These non-specific bindings are weaker than those of the MIP. The difference in binding to the MIP and the NIP are caused by selective binding sites in MIP created by imprinting of template molecule^50^. On the surface of NIP, monomer self-association occurs and reduces the number of free functional groups where the analyte can be bound. Therefore NIP is able to bound less amount of analyte.

**Figure 5:**
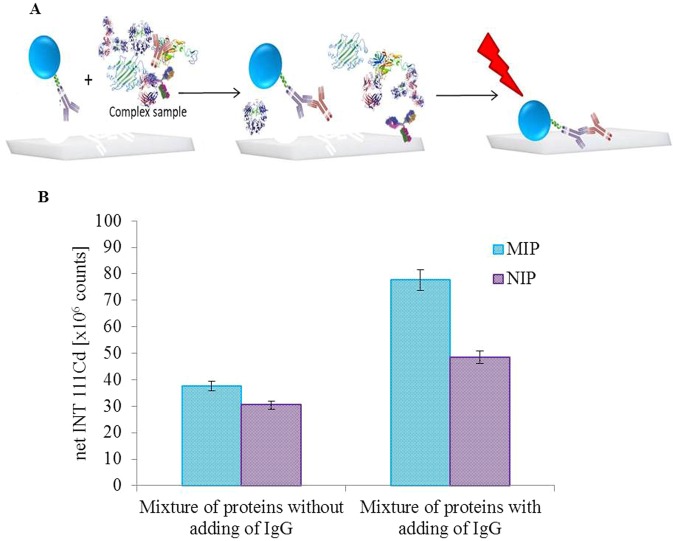
(**A**) Schematic representation of isolation and detection IgG from the same complex sample. At first the conjugate (QD-Ab) is added to the complex sample. The conjugate reacts with IgG contained in sample and creates the complex (QD-AB-AG) with them. The complex is bound to the cavity in MIP that is specific for this. The bound complex is subsequently detected by LA-ICP-MS. (B) ICP-MS signal intensities obtained by analysis of model protein mixture either with or without added IgG extracted by the assay schematically shown in (**A**).

Based on the above mentioned facts, concept B was evaluated as more sensitive as it yields in lower LOD and therefore, was used for isolation and detection of IgG from complex model sample. For this purpose, the protein ladder was used. It consists of mixture of proteins in range from 10–180 kDa (lysozyme, α-lactalbumin, trypsin inhibitor, carbonic anhydrase, ovalbumin, bovine serum albumin, phosphorylase B, β-galactosidase; 0.1–0.3 mg/ml each). Scheme of experiment is illustrated in the Fig. 5A. The idea of this experiment is that the conjugate QD-AB-AG (QD-antiIgG-IgG) is imprinted. Then the QD-AB (QD-antiIgG) conjugate is added into the solution of protein ladder and the AG (IgG) from the sample is captured forming QD-AB-AG (QD-antiIgG-IgG) conjugate. After the overlay of the MIP by this solution, QD-antiIgG-IgG conjugate is captured in the cavities and interfering proteins are washed.

Mixture of proteins with and without addition of IgG was measured. The addition of IgG was 2.5 μg. The mixture of proteins and the QD-AB conjugate was mixed in ratio 1:1. The results are shown in Fig. 5B. It is clearly seen that mixture without IgG addition produces the signal intensity indistinguishable from the NIP, which is due to the nonspecific adsorption. However, it is impossible to distinguish whether the sample components are or the QD-AB conjugate are nonspecifically adsorbed on MIP surface. While after the addition of IgG a significant increase of Cd intensity relating to presence of QD-antiIgG-IgG conjugate is observed. It means that the Concept B is able to isolate IgG from the complex sample containing a mixture of proteins. In comparison results for concept B from Fig. 5 significantly lower sensitivity and higher LOD was found. This deterioration of the parameters is caused probably due to the matrix effect of the proteins present in the sample.

## Conclusions

The MIP technology is currently experiencing a rapid development due to the limitations of natural recognition elements such antibodies or aptamers. However, neither MIP strategies are limitless. Therefore, the combination of these powerful tools in a specific immunoassay may bring highly selective approach. In this work, a MIP-based pseudo-immunoassay using NP-labelled antibody recognition was introduced and coupled with the sensitive detection technique – LA-ICP-MS. Two approaches of specific recognition were tested. The first one was based on the immunolabelling of the analyte captured by the MIP layer. The second approach involved immunolabelling of the analyte as a first step and the resulting QD-AB-AG complex was captured by MIP and further analyzed.

The double-selective approach comprising of the specific immunolabelling reaction combined with isolation by MIP together with the LA-ICP-MS detection represents a viable approach of the IgG detection from a complex sample (LOD 4.2 μg and 1.6 μg, respectively) available for many exciting applications. Considering the overall time of the LA-ICP-MS analysis not exceeding 23 s (scan speed of 2000 μm/s), LA-ICP-MS is a promising technology to be used in future in conjunction with MIP technology.

## Supplementary information

Supplementary information

## References

[1] Vlatakis G, Andersson LI, Muller R, Mosbach K (1993). Drug assay using antibody mimics made by molecular imprinting. Nature.

[2] Wulff G (1995). Molecular imprinting in cross-linked materials with the aid of molecular templates - a way towards artificial antibodies. Angew. Chem.-Int. Edit. Engl..

[3] Baggiani C, Anfossi L, Giovannoli C (2007). Solid phase extraction of food contaminants using molecular imprinted polymers. Anal. Chim. Acta.

[4] Ramstrom O, Skudar K, Haines J, Patel P, Bruggemann O (2001). Food analyses using molecularly imprinted polymers. J. Agric. Food Chem..

[5] Manesiotis P, Borrelli C, Aureliano CSA, Svensson C, Sellergren B (2009). Water-compatible imprinted polymers for selective depletion of riboflavine from beverages. J. Mater. Chem..

[6] Zhu QF, Ma C, Chen HX, Wu YQ, Huang JL (2014). A molecular imprint-coated stirrer bar for selective extraction of caffeine, theobromine and theophylline. Microchim. Acta.

[7] Watabe Y (2005). LC/MS determination of bisphenol A in river water using a surface-modified molecularly-imprinted polymer as an on-line pretreatment device. Analytical and Bioanalytical Chemistry.

[8] Peng Y (2010). Molecularly imprinted polymer layer-coated silica nanoparticles toward dispersive solid-phase extraction of trace sulfonylurea herbicides from soil and crop samples. Anal. Chim. Acta.

[9] Vaneckova T (2019). Molecularly imprinted polymers coupled to mass spectrometric detection for metallothionein sensing. Talanta.

[10] Haupt K, Mosbach K (2000). Molecularly imprinted polymers and their use in biomimetic sensors. Chem. Rev..

[11] Emir Diltemiz Sibel, Keçili Rüstem, Ersöz Arzu, Say Rıdvan (2017). Molecular Imprinting Technology in Quartz Crystal Microbalance (QCM) Sensors. Sensors.

[12] Shepherd RE (2003). Chromatographic and related electrophoretic methods in the separation of transition metal complexes or their ligands. Coord. Chem. Rev..

[13] Lynge ME, van der Westen R, Postma A, Stadler B (2011). Polydopamine-a nature-inspired polymer coating for biomedical science. Nanoscale.

[14] Lee H, Dellatore SM, Miller WM, Messersmith PB (2007). Mussel-inspired surface chemistry for multifunctional coatings. Science.

[15] Liu R, Sha M, Jiang SS, Luo J, Liu XY (2014). A facile approach for imprinting protein on the surface of multi-walled carbon nanotubes. Talanta.

[16] Ryu JH, Messersmith PB, Lee H (2018). Polydopamine Surface Chemistry: A Decade of Discovery. ACS Appl. Mater. Interfaces.

[17] Jia XP (2013). Polydopamine-based molecular imprinting on silica-modified magnetic nanoparticles for recognition and separation of bovine hemoglobin. Analyst.

[18] Xia ZW (2013). Facile synthesis of polydopamine-coated molecularly imprinted silica nanoparticles for protein recognition and separation. Biosens. Bioelectron..

[19] Zhang M, Zhang XH, He XW, Chen LX, Zhang YK (2012). A self-assembled polydopamine film on the surface of magnetic nanoparticles for specific capture of protein. Nanoscale.

[20] Lin ZA (2013). Preparation of boronate-functionalized molecularly imprinted monolithic column with polydopamine coating for glycoprotein recognition and enrichment. J. Chromatogr. A.

[21] Yin YL, Yan L, Zhang ZH, Wang J (2015). Magnetic molecularly imprinted polydopamine nanolayer on multiwalled carbon nanotubes surface for protein capture. Talanta.

[22] Klos-Witkowska A (2016). The phenomenon of fluorescence in immunosensors. Acta Biochim. Pol..

[23] Fu XL, Chen LX, Choo J (2017). Optical Nanoprobes for Ultrasensitive Immunoassay. Analytical Chemistry.

[24] Fan AP, Cao ZJ, Li HA, Kai M, Lu JZ (2009). Chemiluminescence Platforms in Immunoassay and DNA Analyses. Anal. Sci..

[25] Hasanzadeh M, Shadjou N, Soleymani J, Omidinia E, de la Guardia M (2013). Optical immunosensing of effective cardiac biomarkers on acute myocardial infarction. Trac-Trends Anal. Chem..

[26] Wei XF (2018). Multiplexed Instrument-Free Bar-Chart SpinChip Integrated with Nanoparticle-Mediated Magnetic Aptasensors for Visual Quantitative Detection of Multiple Pathogens. Analytical Chemistry.

[27] Sanjay, S. T., Dou, M. W., Sun, J. J. & Li, X. J. A paper/polymer hybrid microfluidic microplate for rapid quantitative detection of multiple disease biomarkers. *Scientific Reports* **6**, 30474 10.1038/srep30474 (2016). 10.1038/srep30474 PMC4960536 27456979

[28] Fu GL, Sanjay ST, Li XJ (2016). Cost-effective and sensitive colorimetric immunosensing using an iron oxide-to-Prussian blue nanoparticle conversion strategy. Analyst.

[29] Fu GL, Sanjay ST, Dou MW, Li XJ (2016). Nanoparticle-mediated photothermal effect enables a new method for quantitative biochemical analysis using a thermometer. Nanoscale.

[30] Buchner T (2016). Biomolecular environment, quantification, and intracellular interaction of multifunctional magnetic SERS nanoprobes. Analyst.

[31] Janu L (2013). Electrophoretic study of peptide-mediated quantum dot-human immunoglobulin bioconjugation. Electrophoresis.

[32] Bilici Mustafa, Zengin Adem, Ekmen Elvan, Cetin Demet, Aktas Nahit (2018). Efficient and selective separation of metronidazole from human serum by using molecularly imprinted magnetic nanoparticles. Journal of Separation Science.

[33] Chen GN (2019). Preparation of molecularly imprinted polymers and application in a biomimetic biotin-avidin-ELISA for the detection of bovine serum albumin. Talanta.

[34] Lai YX (2018). Molecular Imprinting Polymers Electrochemical Sensor Based on AuNPs/PTh Modified GCE for Highly Sensitive Detection of Carcinomaembryonic Antigen. J. Biomed. Nanotechnol..

[35] Li YX, Jiang CY (2018). Trypsin electrochemical sensing using two-dimensional molecularly imprinted polymers on 96-well microplates. Biosens. Bioelectron..

[36] Babamiri B, Salimi A, Hallaj R (2018). A molecularly imprinted electrochemiluminescence sensor for ultrasensitive HIV-1 gene detection using EuS nanocrystals as luminophore. Biosens. Bioelectron..

[37] Ge SG, Lu JJ, Ge L, Yan M, Yu JH (2011). Development of a novel deltamethrin sensor based on molecularly imprinted silica nanospheres embedded CdTe quantum dots. Spectrochimica Acta Part a-Molecular and Biomolecular Spectroscopy.

[38] Jia MF (2017). A molecular imprinting fluorescence sensor based on quantum dots and a mesoporous structure for selective and sensitive detection of 2,4-dichlorophenoxyacetic acid. Sensors and Actuators B-Chemical.

[39] Li JH (2018). Thermosensitive molecularly imprinted core-shell CdTe quantum dots as a ratiometric fluorescence nanosensor for phycocyanin recognition and detection in seawater. Analyst.

[40] Liu YX, Liu L, He YH, He QH, Ma H (2016). Quantum-dots-encoded-microbeads based molecularly imprinted polymer. Biosens. Bioelectron..

[41] Wang XY (2018). Quantum dots based imprinting fluorescent nanosensor for the selective and sensitive detection of phycocyanin: A general imprinting strategy toward proteins. Sensors and Actuators B-Chemical.

[42] Xu SF (2013). Dummy Molecularly Imprinted Polymers-Capped CdTe Quantum Dots for the Fluorescent Sensing of 2,4,6-Trinitrotoluene. ACS Appl. Mater. Interfaces.

[43] Yu JL (2017). One-pot synthesis of a quantum dot-based molecular imprinting nanosensor for highly selective and sensitive fluorescence detection of 4-nitrophenol in environmental waters. Environmental Science-Nano.

[44] Zhang Z, Li JH, Wang XY, Shen DZ, Chen LX (2015). Quantum Dots Based Mesoporous Structured Imprinting Microspheres for the Sensitive Fluorescent Detection of Phycocyanin. ACS Appl. Mater. Interfaces.

[45] Tang YW (2017). A NIR-responsive up-conversion nanoparticle probe of the NaYF4: Er,Yb type and coated with a molecularly imprinted polymer for fluorometric determination of enrofloxacin. Microchim. Acta.

[46] Wang Y (2017). A label-free detection of diethylstilbestrol based on molecularly imprinted polymer-coated upconversion nanoparticles obtained by surface grafting. RSC Adv..

[47] Nejdl L (2018). Rapid preparation of self-assembled CdTe quantum dots used for sensing of DNA in urine. New J. Chem..

[48] http://www.nenovision.com/.

[49] Zhang J, Men YW, Lv SS, Yi L, Chen JF (2015). Protein tetrazinylation via diazonium coupling for covalent and catalyst-free bioconjugation. Org. Biomol. Chem..

[50] Zhou WH (2010). Mussel-inspired molecularly imprinted polymer coating superparamagnetic nanoparticles for protein recognition. J. Mater. Chem..

