# Supplementary material for: CdS quantum dots-based immunoassay combined with particle imprinted polymer technology and laser ablation ICP-MS as a versatile tool for protein detection
Source: Sci Rep. 2019 Aug 14;9:11840. doi: 10.1038/s41598-019-48290-2 (PMC6694198; doi:10.1038/s41598-019-48290-2)
Supplement: Supplementary file 1 — Supplementary information [file 41598_2019_48290_MOESM1_ESM.docx]

Supplementary material

# CdS quantum dots-based immunoassay combined with particle imprinted polymer technology and laser ablation ICP-MS as versatile tool for protein detection

Tereza Vaneckova^1,2^, Jaroslava Bezdekova^1,2^, Michaela Tvrdonova^4^, Marcela Vlcnovska^1,2^, Veronika Novotna^3^, Jan Neuman^3^, Aneta Stossova^4^, Viktor Kanicky^4,5^, Vojtech Adam^1,2^ , Marketa Vaculovicova^1,2*^ and Tomas Vaculovic^4,5^

^1^ Department of Chemistry and Biochemistry, Mendel University in Brno, Zemedelska 1, CZ-613 00 Brno, Czech Republic

^2^ Central European Institute of Technology, Brno University of Technology, Purkynova 123, CZ-612 00 Brno, Czech Republic

^3^ NenoVision s.r.o., Purkynova 649/127, CZ-612 00, Brno, Czech Republic

^4^ Department of Chemistry, Masaryk University, Kamenice 753/5, CZ-625 00 Brno, Czech Republic
^5^ Central European Institute of Technology, Masaryk University, Kamenice 753/5, CZ-625 00 Brno, Czech Republic

Corresponding Author

* Marketa Vaculovicova, Department of Chemistry and Biochemistry, Mendel University in Brno, Zemedelska 1, CZ-613 00 Brno, Czech Republic, [marketa.ryvolova@seznam.cz](mailto:marketa.ryvolova@seznam.cz)

**S1**

**DLS characterization of CdS quantum dots. A) size, B) zeta potential**

**A)**

**B)**

**S2**

**TEM micrograph of CdS quantum dots, scale bar = 50 nm**

**
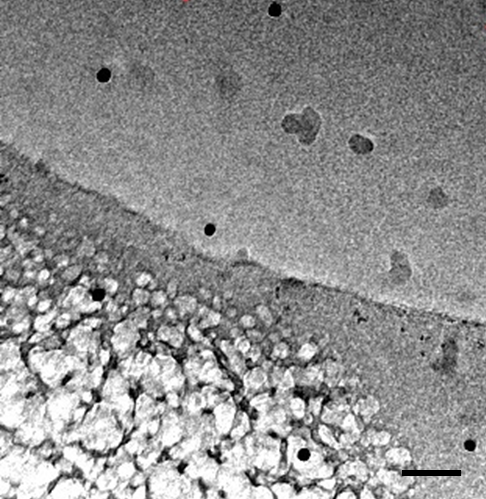
**

**S3**

**TEM micrograph of CdS quantum dots – antibody conjugate (red arrow), scale bar = 50 nm**

**
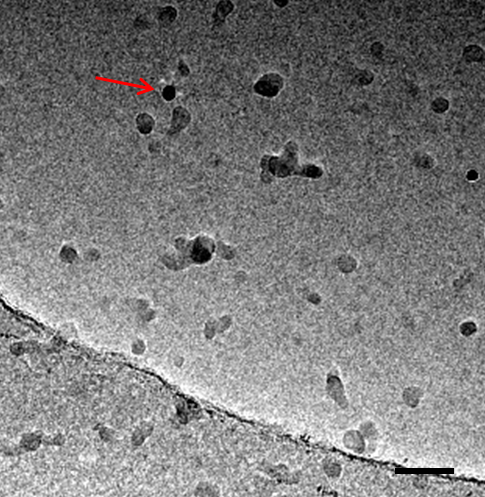
**
